# Bird immunobiological parameters in the dissemination of the biofilm-forming bacteria *Escherichia coli*

**DOI:** 10.14202/vetworld.2023.1052-1060

**Published:** 2023-05-17

**Authors:** Ekaterina Lenchenko, Nadezhda Sachivkina, Tatiana Lobaeva, Natallia Zhabo, Marina Avdonina

**Affiliations:** 1 Department of Veterinary Medicine, Russian Biotechnological University (BIOTECH University), 125080, Moscow, Russia; 2 Department of Microbiology V.S. Kiktenko, Institute of Medicine, Peoples’ Friendship University of Russia named after Patrice Lumumba (RUDN University), 117198, Moscow, Russia; 3 Department of Biochemistry T.T. Berezov, Institute of Medicine, Peoples’ Friendship University of Russia named after Patrice Lumumba (RUDN University), 117198 Moscow, Russia; 4 Department of Foreign Languages, Institute of Medicine, Peoples’ Friendship University of Russia named after Patrice Lumumba (RUDN University), 117198, Moscow, Russia; 5 Department of Linguistics and Intercultural Communication, Moscow State Linguistic University, 119034, Moscow, Russia

**Keywords:** avian colibacillosis, biofilm formation, *Escherichia coli*, intercellular matrix, poultry

## Abstract

**Background and Aim:**

With the development of industrial maintenance technology, a group of pathogens called avian pathogenic *Escherichia coli* (APEC) became very common. The initiation, development, and outcome of the infectious process mediated by virulent APEC strains occur through a decrease in the colonization resistance of the intestine, an immunobiological marker of homeostasis stability in susceptible species. This study focused on the pathogenetic features of colibacillosis and the morphological features of *E. coli*.

**Materials and Methods:**

Clinical, immunological, bacteriological, and histological studies were conducted on 15-day-old white Leghorn birds (n = 20). The birds were divided into two groups: Control group (Group I; n = 10) with birds intranasally inoculated with 0.5 mL of 0.9% NaCl solution and experimental group (Group II; n = 10) with birds intranasally inoculated with 0.5 mL of an *E. coli* suspension at 1 billion/mL.

**Results:**

During the biofilm formation, clusters of microcolonies were formed as a gel-like intercellular matrix that accumulated due to cell coagulation. The intercellular matrix “glues” heteromorphic cells together and forms a structure of densely packed heteromorphic cells arranged in an orderly manner and growing in different directions. During the experimental reproduction of *E. coli*, excessive growth was observed in material isolated from poultry. Pathogenic *E. coli* strains implementing virulence factors adhered to the receptors of erythrocytes, alveolocytes, and enterocytes. Multicellular heterogeneous biofilms, united by an intercellular matrix, were located at the apical poles of the respiratory tract alveolocytes and enterocytes of the terminal ileum villi. Many bacteria exudate containing desquamated epithelial cells with an admixture of mucus, and polymorphonuclear leukocytes were detected in the lumen of the birds’ abdominal organs. Invasive bacteria damaged the epithelial layer, violated the endothelial layer of blood vessels, and developed inflammatory hyperemia of the lamina propria of the respiratory and digestive systems’ mucous membrane. A correlative dependence of changes developed by the type of delayed hypersensitivity reaction was established. Signs of accidental transformation of the thymus, atrophy of the bursa of Fabricius, disseminated thrombosis, and septic spleen developed. Moreover, toxic cardiomyocyte dystrophy, signs of congestive vascular hyperemia, massive disintegration of lymphocytes, macrophage reactions, perivascular edema resulting from the release of plasma, and shaped blood elements were detected.

**Conclusion:**

The development and outcome of the infectious process in escherichiosis primarily depend on the homeostasis stability of susceptible species and virulence factors of the pathogenic microorganisms. One of the selected strains, *E. coli* O78:K80 displayed the highest ability to form biofilms. Its strong adhesion ability to bird erythrocytes was demonstrated. Deepening the scientific knowledge of the interaction between eukaryotes and prokaryotes will contribute to a better understanding of the pathogenetic aspects of avian escherichiosis and eventually find promising anti-adhesive drugs that could reduce primary bacterial contamination *in vivo* and *in vitro*.

## Introduction

Domestication in an unnatural habitat, limited territorial space, and unconventional feeding are the leading causes of the development of pathological processes in wild, exotic, and ornamental birds [1, 2]. A group of pathogens called avian pathogenic *Escherichia coli* (APEC) are the most common among the bacterial agents found in industrial maintenance technology [3–5]. In serological identifications of escherichiosis pathogens in chickens, turkeys, and other bird species, the *E. coli* serotypes O1, O2, and O78 are most often isolated [6–8]. Identifying isolates producing adhesive antigens, *E. coli* O1:K99, O1:F41, O1:K99:F41, O2:K88, O2:K99:F41, O78:K88, O78:K99, and O15:K88:F41, accompanied by the development of aerosacculitis, explained generalized infection and septicemia [9]. When susceptible species are infected, the bacteria release virulence factors encoded by plasmid genes in bacteriophages [1, 10, 11]. Based on the virulence gene profiles, pathogenic *E. coli* are divided into several pathotypes: Enterohemorrhagic *E. coli*; 9.3%, enteropathogenic *E. coli*; 39.5%, enteroaggregative *E. coli* (EAEC; 1.2%), enteroinvasive *E. coli*, enterotoxigenic *E. coli* (ETEC; 5.8%), diffusely adherent *E. coli*, and APEC; 32.53% [12, 13]. The lower percentage of EAEC and ETEC compared to other pathotypes is statistically significant. Several factors have been associated with the virulence of *E. coli* in avian hosts, and only three isolates were identified as O157 based on detecting the *rbfO15*7 gene [14]. Of the 2200 *E. coli* isolates from samples of clinically healthy commercial meat chickens, 751 (34.0%) were classified as APEC with 4.9 virulence genes (VG) per isolate, and 1449 (66.0%) were *E. coli* APEC with 2.2 VG/isolate [15].

The phenotypic plasticity of microorganisms contributes to their long-term persistence and the expansion of their adaptive potential, which determines the formation of microbial biofilms as protection from immune responses and the effects of chemotherapeutic drugs and disinfectants [11, 16]. *Escherichia coli* strains (n = 86) were isolated from cloaca flushes and avian material and were found resistant to ampicillin (75.6%), gentamicin (39.5%), and tetracycline (29.1%). However, the isolates were sensitive to imipenem [14]. *Escherichia coli* strains (n = 717) isolated from poultry and environmental samples displayed resistance to tetracycline (91.2%), oxytetracycline (89.1%), sulfamethoxazole/trimethoprim (73.1%), doxycycline (63.0%), and sulfamethoxazole (63.0%) [17]. *Escherichia coli* isolates producing broad spectrum β-lactamases were more often isolated from samples of broiler chickens, which are a reservoir of multidrug-resistant bacteria [18].

The initiation, development, and outcome of the infectious process mediated by virulent strains occur through a decrease in the colonization resistance of the intestine, which is an immunobiological marker of the homeostasis stability of susceptible species [19–22].

This study aimed to determine the immunobiological parameters of birds during the experimental reproduction of an infectious process mediated by biofilm-forming bacteria. The approval and search for efficient methods to study the interaction mechanism between eukaryotes and prokaryotes can potentially improve long-term and retrospective bacteriological diagnostic schemes. They will contribute to developing drugs with antigenic and immunogenic activity, a broad spectrum of action, and universal for animals and birds from different climatic zones. Such studies offer a perspective for developing effective anti-adhesive chemotherapeutic and disinfecting drugs. In addition to the application aspects, priority tasks of general pathology can be solved by understanding the pathogenetic mechanisms of microorganisms’ adaptation to long-term persistence *in vivo* and *in vitro*.

## Materials and Methods

### Ethical approval

This study complied with the requirements of the “Directive 2010/63/EU of the European Parliament and the Council of the European Union” (September 22, 2010) on the protection of animals used for scientific purposes. All bird experiments followed the Guide for the Care and Use of Laboratory Animals [23] (Committee for the Update). Birds were manipulated following the Local Ethics Committee for Animal Experimentation, Shared Research and Educational Center of RUDN University (protocol number 73; September 10, 2022).

### Study period and location

The study was conducted from September 1, 2022, to September 20, 2022, at the experimental base of Shared Research and Educational Center of RUDN University.

### Bacterial strain

The reference strain of *E. coli* ATCC 25922 used in this study was obtained from the State Research Institute for Standardization and Control of Medical Biological Preparations collection, named after L.A. Tarasevich (Moscow) [24]. We used the isolates O2:A20 and O78:K80 obtained from white Leghorn birds with septicemia [25]. Microorganism cultures were stored in semi-liquid 0.5% of meat-peptone agar in the freeze-dried form at 4°C ± 1°C.

### Bacterial phenotypes

Microorganisms’ morphological, cultural, and chemical properties were studied using conventional methods with differential diagnostic media and test systems. The bacterial phenotypes were studied using conventional methods according to the classification system from Bergeys manual 1984–1989 [26]. Microorganisms were cultured at 37°C ± 1°C for 24 or 48 h on the following media: Blood Agar Base (Biomerieux, France), Nutrient Broth for the general cultivation of less fastidious microorganisms (HiMedia, India), Endo Agar (HiMedia), Tryptone Bile X-glucuronide agar (Merck, Germany), and Chromogenic *E. coli*/coliform agar (Merck). The ENTERO-Rapid 24 kit (PLIVA-Lachema, Czech Republic) was used to differentiate microorganisms. Serological identification was conducted at the Federal Budgetary Institution of Science “State Scientific Center for Applied Microbiology and Biotechnology” https://obolensk.org/en/about-eng (Russian Federation) using the “E-coli serum” and “Anti-adhesive serums”, prepared at this Institution and on “Armavir Biofactory” (Russian Federation).

### Morphometric and densitometric indicators of bacterial biofilms

The morphometric and densitometric indicators of bacterial biofilms were studied using flat-bottomed, sterile, 12-well culture plates (Medpolymer, Russia) with lids with a treated surface for monolayer cell cultures [27]. The wells had a volume of 6.8 mL, and small glasses (18.0 × 18.0 mm; Corning Inc., USA) were placed at the bottom of the holes for microbiological studies. Later, 3.0 mL of Nutrient Broth and 1.0 mL of a bacterial suspension at a concentration of 0.5 units (McFarland) were placed in the wells. Microorganisms were cultured for 24 and 48 h at 37°C ± 1°C. The plates were mixed at 2000 rpm for 10 min using a MixMate vortex shaker (Eppendorf, Germany) before and after cultivation. Biofilm preparations were fixed on the glass surface with a mixture of alcohol and ether (1:1) for 10 min. For optical microscopy, the preparations were stained with an aqueous solution of gentian violet (dilution 1:2000; HiMedia) and a coloring Gram-staining kit (BioVitrum, Russia).

Morphometric studies were conducted on a representative sample with a reliable occurrence frequency ≥90.0% in the field of view of the optical microscope Trinocular Unico (Unico, USA). The electron microscopy analysis was performed using a Hitachi TM4000Plus (Hitachi, Japan), and the samples were sputtered with Q150T ES gold ions (Quorum Technologies, Great Britain).

The optical density of biofilms was determined by the binding degree of crystal violet (HiMedia) using an Immunochem–2100 microplate photometric analyzer (HTI, USA) at 490 nm. The test material was fixed with 150.0 μL of 96.0% ethanol for 15 min before drying at 37°C ± 1°C for 20 min. The well contents were removed, washed 3 times with 200.0 μL of phosphate buffer solution (pH = 7.3), and dried. Then, 200 μL of 0.5% dye solution was added to the wells, and the whole was cultured for 5 min at 37°C ± 1°C. The bound dye was eluted from adherent cells with 96.0% ethanol for 30 min.

For weak biofilm producers, the optical density of the sample or the microorganism culture (sample density [Ds]) is < 2 times (Ds ≤ 0.196) higher than the control optical density of the control (control density [Dc]) (i.e., nutrient medium without inoculum). Sample densityexceeds Dc by 2–4 times (Ds = 0.196–0.392) for moderate biofilm producers. Finally, for strong biofilm producers, Ds exceeds Dc by more than four times (Ds ≥ 0.392). Optical and scanning electron microscopy samples were prepared using standard methods [10, 11, 28].

### Experimental animals

The study was conducted on clinically healthy birds (n = 20) of the white Leghorn. The experiment lasted for 20 days.

### Bird keeping

The birds were kept in cages with the following dimensions: Length of 710.0 cm, width of 260.0 cm, and height of 200.0 cm. The cage floor was made of metal rods, and the maximum load was 90.0 kg. A feeder with a lid provided an economical and even feed quantity, and limiter windows prevented the spread of feed inside the cage. The feed comprised corn, soybean meal, sunflower meal, protein feed mixture, limestone flour, monophosphate, fish flour, lysine, table salt, methionine, and baking soda. The water drinkers were inside the cages — the nipple watering system does not allow water to spill, so the cage always remained dry. The cages had a mechanical litter removal system allowing easy and efficient removal of litter and debris. After turning the handle, all the droppings fall into a tray, easily removed, and washed. Throughout the experiment, food and water were provided without restrictions. The ambient temperature was 20°C ± 1°C, and the relative humidity was 58% ± 1.0%.

### Bacterial adhesive properties

Blood was taken from the axillary vein from the inside of the wing to study the adhesive properties of bacteria. The blood collection area, closer to the elbow joint, was cleaned of small feathers and wiped with a cotton swab moistened with 70% ethanol. The puncture was made on the left of the breast bone in a V-shaped cut with the needle’s direction toward the shoulder joint of the opposite side. 2.0 mL of blood and 0.1 mL of 2.0% sterile sodium citrate solution were injected into 5.0 mL test tubes (Eppendorf). Then, 0.5 mL of *E. coli* bacterial suspension (1 billion/mL) inactivated at 60°C for 30 min was added to the test tubes. Samples were homogenized using the RM 100 Touch mixer (Lamy Rheology, France) and cultured at 37°C ± 1°C for 15 and 120 min [29].

### Experimental infection of birds

Fifteen day-old female birds (n = 20) were divided into two groups according to the analog principle (same age, sex, live weight, feeding conditions, and maintenance). In the first control group (Group I; n = 10), birds were intranasally inoculated with 0.5 mL of 0.9% NaCl solution. In the second experimental group (Group II; n = 10), birds were intranasally inoculated with 0.5 mL of an *E. coli* suspension at 1 billion/mL.

### Investigation of bacterial dissemination

To isolate and identify pure cultures of *E. coli*, feces and pathological material, a heart with ligated vessels, a tubular bone, a liver with a gallbladder, and an affected area of the small intestine tied with a ligature were examined. From the pathological material, crops were prepared with a Pasteur pipette on a medium surface in Petri dishes and were evenly rubbed with a glass spatula. Organ crops, except the small intestine, were also prepared using “prints” of the cut surface of an organ piece from a pre-profiled area on a nutrient medium in Petri dishes. The contents were removed for the small intestine examination. The intestinal mucosa was scraped off with the scarifying cone of a Pasteur pipette and applied to the medium surface in Petri dishes. Before sowing the material, the surface of the nutrient media was irrigated with 1–2 cm^3^ of 96° ethyl alcohol to delay the growth of the swarming form of *Proteus* bacteria [29, 30].

### Histopathological analysis

Samples for histological studies were placed in 10.0% neutral formalin and filled with paraffin. Histological sections were stained with hematoxylin and eosin, and immunobiological parameters were measured by optical microscopy following standard histological procedures [14, 31].

### Statistical analysis

Experimental data were processed using descriptive and logical statistics. The average values and standard deviations of optical densities, bacterial adhesive properties of bacteria, and phagocytic activity of hemocytes were calculated using Microsoft Excel. The difference between the mean samples and the control values was determined using Student’s t-test, and the statistical significance of the differences was established at p ≤ 0.05.

## Results

### Bacterial phenotypes

**Figure-1:**
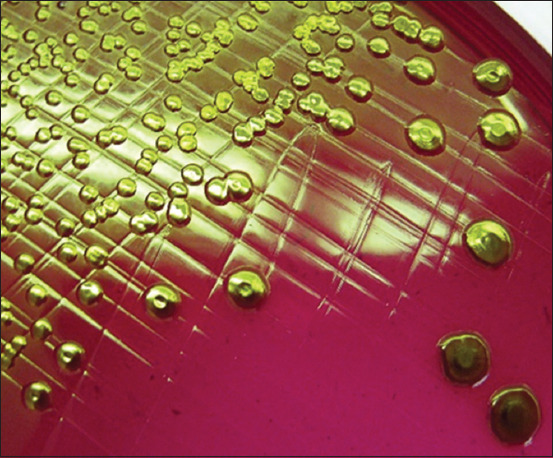
Morphology of *Escherichia coli* О78:К80 colonies, “Agar Endo,” 37°C ± 1°C, 24 h.

**Figure-2:**
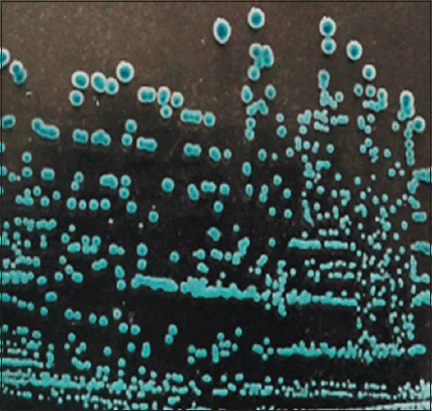
Morphology of *Escherichia coli* О78:К80 colonies, “Tryptone Bile X-glucuronide agar,” 37°C ± 1°C, 24 h.

**Figure-3:**
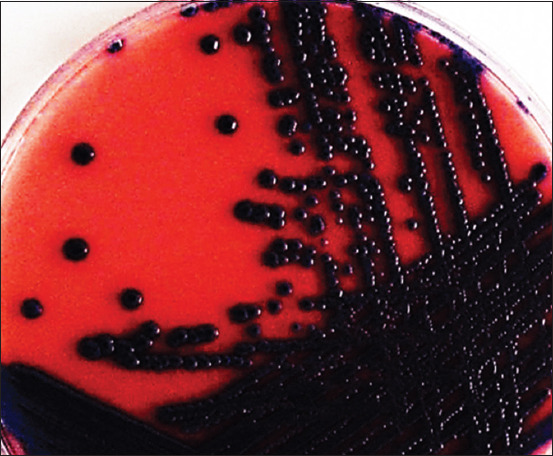
Morphology of *Escherichia coli* О78:К80 colonies, “Chromogenic *E. coli*/Coliform medium agar,” 37°C ± 1°C, 24 h.

Cultures of *E. coli* at 37°C ± 1°C for 24 h under aerobic and anaerobic conditions formed colonies with typical color and shape on the surface of different diagnostic media. Lactose-fermenting *E. coli* formed red colonies with metallic luster on Endo Agar (Figure-1). β-glucuronidase-fermenting *E. coli* formed blue colonies on Tryptone Bile X-glucuronide agar (Figure-2). The bacteria formed purple colonies on the Chromogenic *E. coli*/coliform medium agar, splitting two chromogenic substrates simultaneously due to the presence of β-galactosidase and β-glucuronidase (Figure-3).

The studied microorganisms were Gram-negative, catalase-positive, and oxidase-negative. They fermented D-glucose and polyatomic alcohols with the formation of acid and gas, formed indole, utilized sodium acetate, did not form hydrogen sulfide, did not utilize citrate or sodium malonate, and did not produce urease or phenylalanine deaminase.

### Morphometric and densitometric indicators of bacterial biofilms

**Figure-4:**
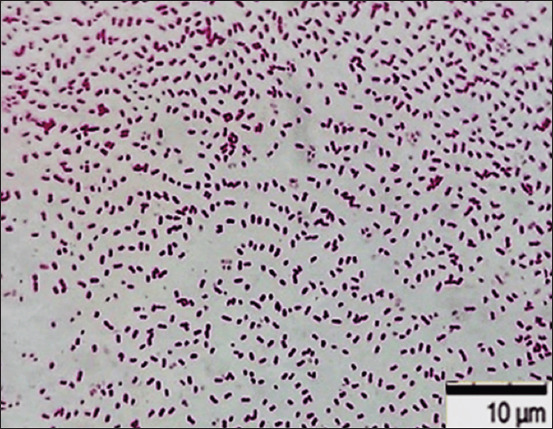
Culture of *Escherichia coli* О78:К80 from poultry patmaterial isolated during septicemia, Nutrient Both, 37°C ± 1°C, 24 h: Gram-negative rod-shaped bacteria. Gram-staining. Magnification: 10 × 100, immersion (H604 Trinocular Unico, USA).

**Figure-5:**
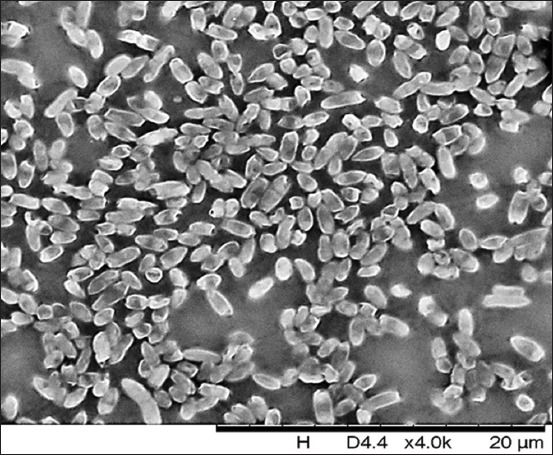
Culture of *Escherichia coli* ATCC 25922, Nutrient Both medium, 37°C ± 1°C, 48 h: densely located bacteria. Scanning electron microscopy. Magnification: 2000× Hitachi TM3030 Plus (Japan).

**Figure-6:**
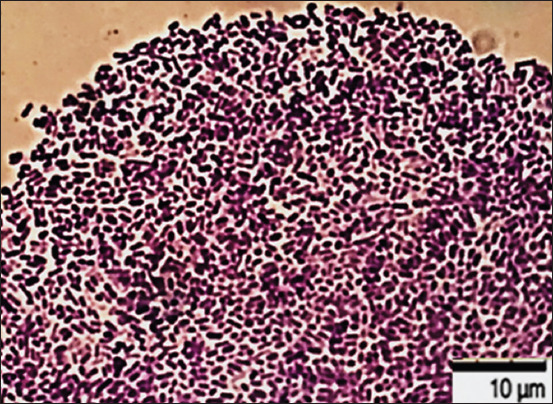
Culture of *Escherichia coli* О2:A20 from poultry patmaterial isolated during septicemia, nutrient both, 37°C ± 1°C, 48 h: Rod-shaped bacteria. Gentian violet staining. Magnification: 10 × 100, immersion (H604 Trinocular Unico, USA).

After 24–48 h of cultivation in a liquid nutrient medium, the Nutrient Broth revealed general patterns of biofilm formation of the studied *E. coli* strains ATCC 25922, O2:A20, and O78:K80. Optical and scanning electron microscopy revealed the following stages of biofilm formation: adhesion, fixation, microcolony, growth, and dispersion. The bacteria displayed a flattened shape during the deposition and adhesion of planktonic cells to the substrate surface. These flattened cells, tightly attached to the substrate surface, contribute to the subsequent attachment of bacteria, usually having a typical rod-shaped shape. At the same time, bacterial cells are detected at the stage of binary division. The architectonics of biofilms rely on synthesizing an intercellular matrix from gel-like exocellular structures. The coagulation of bacterial cells united by the intercellular matrix appears in short and long chains, and a diffuse monolayer of bacterial cells is gradually formed (Figures-4 and 5). As the gel-like intercellular matrix accumulates, clusters of microcolonies form due to cell aggregation. Microcolonies, structural and functional biofilm units represent the immobilization of the bacterial population. The intercellular matrix “glues” heteromorphic cells together and forms a dense structure of heteromorphic cells arranged in an orderly manner and spreading in different directions (Figure-6).

Microcolonies are a hydrated population of heterotrophic microorganisms that resemble macroscopic colonies on dense agar media (Figures-1–3). Pores and tubules of rounded shape, characterized by the presence of fluid and surrounded by membrane structures, were revealed on the lines of cluster formation. This observation corresponds to population growth, characterized by the accumulation of nutrient-containing fluid. The “mature” biofilm changes size and shape and the intercellular matrix performs a protective function.

As a certain optical density of the intercellular matrix was reached, a tendency for differences between the central and peripheral parts of the colonies was observed. The initial organization level of the central part of the microcolonies was maintained as densely arranged typical rod-shaped cells without significant changes in the structure of the intercellular matrix. At the same time, the intercellular matrix of the microcolonies’ peripheral sections was thinned, and the degree of compactness of heteromorphic cells changed significantly. This stage corresponds to the dispersion of planktonic forms of the population; heteromorphic structures with cell wall defects were observed, while typical rod-shaped bacteria preserved the integrity of their cell wall producing the intercellular matrix. Due to the adhesion of the latter, new secondary microcolonies were formed, separated from the original “mother” microcolony by matrix voids. Due to long filamentous structures — cellular strands — a “movement” of the mucous gel-like structures of the bacterial population was observed. This is how the colonization of remote “uninhabited” parts of the substrate and the d destruction of the “mother” microcolony gradually occur.

Densitometric indicators of the samples were: Ds *E. coli* ATCC 25922 = 0.338 ± 0.27; Ds *E. coli* O2:A20 = 0.471 ± 0.34; Ds *E. coli* O78:K80 = 0.511 ± 0.11, and Dc = 0.98 ± 0.09. The analysis of the densitometric indicators indicated that *E. coli* O78:K80 was the strongest biofilm producer. Therefore, the rest of the experiments were conducted on this serotype.

### Bacterial adhesive properties

**Figure-7:**
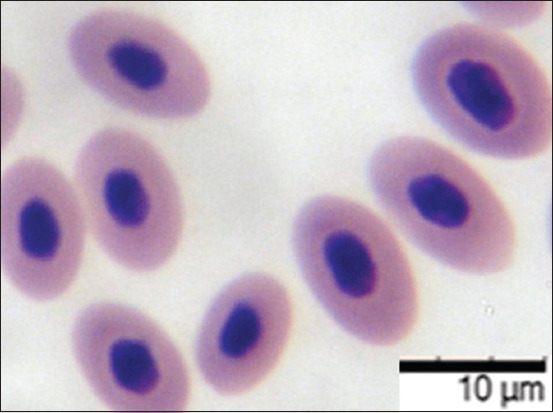
Blood smear of bird ♀, 37°C ± 1°C, 24 h. May-Grunwald staining, optical microscopy, 900×, immersion (“H604 Trinocular Unico,” USA).

**Figure-8:**
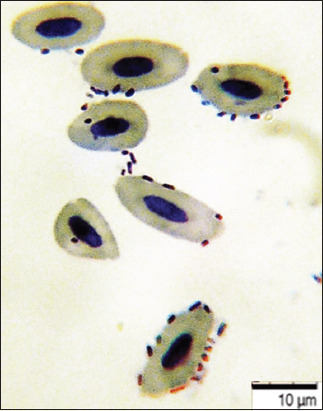
Blood smear of bird ♀, 22°C ± 1°C, 24 h: Interaction of blood cells and *Escherichia coli* bacteria. May-Grunwald staining, optical microscopy, 900×, immersion (“H604 Trinocular Unico,” USA).

The study of the interaction of bacteria and avian blood cells revealed common patterns of interaction between eukaryotes and prokaryotes. Working with the red blood cells of birds, we observed the same processes as with the blood cells of fish and mammals, as previously described [31–33]. When interacting with bird blood erythrocytes, the adhesion index of *E. coli* O78:K80 was 4.33 ± 0.09, corresponding to high adhesion. Due to the presence of fimbrial structures and fimbrial adhesins, the pathogenic bacteria adhered well to the receptors of the avian red blood cells (Figures-7 and 8).

### Histopathological studies of the dissemination of *E. coli* O78:K80 into the bird tissues and organs

The intranasal infection of birds with *E. coli* O78:K80 led to bacterial dissemination into the tissues and organs of the birds. After 5–6 days, *E. coli* colonies were isolated from the birds’ blood, lungs, small intestine, liver, and kidneys. After 11–12 days, *E. coli* colonies were isolated from the blood, spleen, and kidneys.

The dynamics of pathological processes during bacterial dissemination were characterized by the bruising of the skin, hyperemia, cyanosis, vascular hemorrhages, fibrinous aerosacculitis, pericarditis, catarrhal fibrinous enteritis, perihepatitis, and multiple kidney hemorrhages. As a rule, serous fibrinous aerosacculitis and pericarditis were observed 4–5 days after infection. Foamy exudate was detected in the trachea, and the lungs displayed a flabby consistency. The air sacs were thickened, dull, and opaque with overlays of fibrin films. Accumulation of catarrhal exudate and serous cell effusion was observed in the alveoli, in addition to hyperemia and leukocyte infiltration and fibroblast proliferation of perivascular connective tissue in the interlobular connective tissue. In the parabronchi cavities, light pink fibrin filamentous structures were revealed, and the bronchi lumen was filled with many pseudoeosinophils and lymphocytes. Spot hemorrhages were detected in the heart membranes, and the pericardium was stretched and filled with serous fibrinous exudate. The epicardium was covered with fibrin films, dull, reddened, and thickened due to impregnation with serous fibrinous exudate and infiltration by pseudoeosinophils, lymphocytes, histiocytes, and fibroblasts. The areas of the myocardium adjacent to the epicardium were infiltrated by pseudoeoinophils, lymphocytes, and histiocytes. The endocardium blood vessels were dilated and filled with densely located erythrocytes and pseudoeoinophils. As a rule, signs of toxic cardiomyocyte dystrophy developed.

Multicellular heterogeneous biofilms, united by an intercellular matrix, were located at the apical poles of the respiratory tract alveolocytes and enterocytes of the terminal ileum villi. Several bacteria, exudates containing desquamated epithelial cells with an admixture of mucus, and polymorphonuclear leukocytes were detected in the lumen of the abdominal organs. Invasive bacteria damaged the epithelial layer, revealed a violation of the endothelial layer of blood vessels, and led to the development of inflammatory hyperemia of the lamina propria of the mucous membrane of the respiratory and digestive systems.

**Figure-9:**
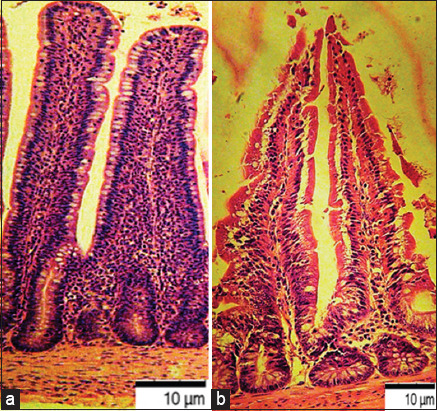
Small intestine of bird ♀ when infected with *Escherichia coli* O78:K80 from poultry patmaterial isolated during septicemia, Nutrient Both, 37°C ± 1°C, 24 h: (a) Control; (b) Experiment: Damage to the epithelial layer, most villi were destroyed, revealed a violation of the endothelial layer of blood vessels, the development of inflammatory hyperemia of the own plate of the mucous membrane.

Pathogenic bacteria adhered to the receptors of villus enterocytes and crypts of the mucous membrane of the terminal small intestine. Invasive bacteria damaged the epithelial layer, destroying most of the villi, violated the endothelial layer of blood vessels, and led to the development of inflammatory hyperemia of the lamina propria of the mucous membrane (Figure-9).

Pathologies caused by the dissemination of microorganisms were characterized by dystrophic and necrotic processes of the hepatobiliary system and the nephrotoxic syndrome manifested by congestive hemorrhagic infarction and necrosis of the epithelium of the tubules of the nephrons. Microabscesses, necrosis of the tubule epithelium, and inflammatory vascular hyperemia were noted in the parenchyma of the cortical and cerebral layers of the kidneys.

**Figure-10:**
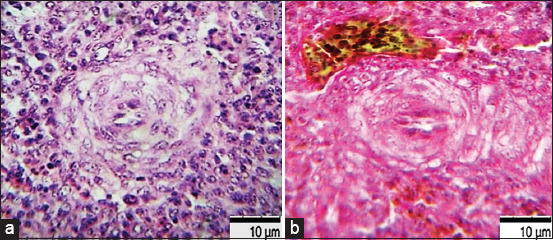
Spleen of bird ♀ when infected with *E. coli* O78:K80 from poultry pathological material isolated during septicemia, Nutrient Both, 37 ± 1°C, 24 h: (a) control (b) experiment: impaired vascular permeability, perivascular edema, periarterial hemorrhages. Hematoxylin and eosin, optical microscopy, 900×, immersion («H604 Trinocular Unico», USA).

A correlative dependence of changes developed by the type of delayed hypersensitivity reaction was established. Signs of accidental transformation of the thymus, atrophy of the bursa of Fabricius, disseminated thrombosis, and septic spleen developed. Signs of congestive vascular hyperemia, massive disintegration of lymphocytes, macrophage reactions, perivascular edema of tissues due to the release of plasma, and shaped blood elements were revealed (Figure-10).

## Discussion

Avian colibacillosis is among the major causes of worldwide economic loss in the poultry industry, with a more vivid impact on developing countries. Our research and analysis of the relevant literature indicated that the pathogenesis of overgrowth syndrome in the translocation of microorganisms and the implementation of virulence factors are provided by adhesive properties [11, 20, 23, 27]. Adhesion is a key factor in forming biofilm architectonics, characterized by increased optical density, determining the duration and retrospective diagnostic studies of microorganisms [16, 22, 29]. Dissociation processes were detected in biofilms of polyresistant strains; densitometric indicators of the optical density of smooth colonies were 20.0%–60.0% lower than other phenotypes [11]. Populations exposed to unfavorable factors were heterogeneous; they did not differ genetically from the original population and retained the physiological diversity necessary to colonize a “purified” niche or a new habitat [11, 26]. To detect viable microorganisms in a heterogeneous population, the effectiveness of instrumental research methods revealing the stages of interaction between eukaryotes and polysaccharides of the cell wall of various microorganism systematic groups has been established [20, 21, 30]. Indicators of biomass growth of the gel-like mucous structure of biofilms were revealed by phase contrast microscopy; viable and non-viable cells were differentiated by luminescent microscopy; and sorption and aggregation of heterogeneous biofilms, cyclic growth modes, and phenotypic plasticity were studied by electron microscopy [11, 21, 31]. To reveal the mechanisms of intercellular communication of monovid and polyvid biofilms and the pathogenetic aspects of the infectious process, methodological approaches have been developed to preserve the natural architectonics of biofilms *in vitro, ex vivo*, and *in vivo* [20, 21, 32, 33]. Considering the direct correlative relationships between the volume of viable cells and the volume of β-polysaccharides of the intercellular matrix, anti-adhesive drugs, protein molecules quorum sensing, and bacteriocins have promising potentials [16, 26, 34–40]. Farnesol is recommended for the negative regulation of hyphae-specific genes (*EFG1*, *CPH1*, and *HST1*) and the depression of transcription repressors (TUP1 and NRG1) [41–45]. All these works partially studied the pathogenetic features of the disease and the features of the pathogen morphology. However, before the present study, no comprehensive study had confirmed the relationship or dependence between the ability to form biofilms or adhere and the pathogenicity of colibacillosis.

Priority areas for scientific research should include expanding our understanding of the mechanisms of pathogen adaptation during parasitism in susceptible species, optimizing the scheme of microbiological research, developing antiepizootic measures to prevent animal and poultry diseases, and obtaining safe food and environmental protection.

## Conclusion

During the formation of biofilms, clusters of microcolonies are formed as the gel-like intercellular matrix accumulates due to cell coagulation. The intercellular matrix “glues” heteromorphic cells and population structures from densely packed heteromorphic cells arranged in an orderly manner but spreading in different directions. The development and outcome of the infectious process in escherichiosis are primarily due to the homeostasis stability of susceptible species and the virulence factors of pathogenic microorganisms. The *E. coli* O78:K80 strain was selected in this study as having the greatest ability to form biofilms. Its strong adhesive ability to bird erythrocytes was demonstrated. The correlative dependence of changes developed by the type of delayed hypersensitivity reaction was established. Signs of accidental transformation of the thymus, atrophy of the bursa of Fabricius, disseminated thrombosis, and septic spleen developed. In addition, signs of congestive vascular hyperemia, massive disintegration of lymphocytes, macrophage reactions, perivascular edema of tissues formed due to the release of plasma, and shaped blood elements were revealed. A deeper study of the interaction of eukaryotes and prokaryotes will contribute to understanding the pathogenetic aspects of avian escherichiosis and support the search for promising anti-adhesive drugs that could reduce primary bacterial contamination *in vivo* and *in vitro*.

## Authors’ Contributions

EL and NS: Conceptualized and designed the study. EL: Collected the samples. TL: Data analysis and data cleaning. NZ and MA: Study design, statistical analysis, and drafted and revised the manuscript. All authors have read, reviewed, and approved the final manuscript.

## Data Availability

All data generated during the study arewithin the published article.
